# Immune Events Associated with High Level Protection against *Schistosoma japonicum* Infection in Pigs Immunized with UV-Attenuated Cercariae

**DOI:** 10.1371/journal.pone.0013408

**Published:** 2010-10-15

**Authors:** Fang Tian, Dandan Lin, Jingjiao Wu, Yanan Gao, Donghui Zhang, Minjun Ji, Guanling Wu

**Affiliations:** 1 Department of Pathogen Biology and Immunology, Nanjing Medical University, Nanjing, Jiangsu, China; 2 Jiangsu Province Key Laboratory of Modern Pathogen Biology, Nanjing, Jiangsu, China; 3 Jiangxi Provincial Institute of Parasitic Diseases, Nanchang, Jiangxi, China; 4 Department of Pathogen Biology and Immunology, Yangzhou University Medical College, Yangzhou, Jiangsu, China; BMSI-A*STAR, Singapore

## Abstract

**Background:**

The vaccination of radiation-attenuated *Schistosoma japonicum* cercariae can induce effective protection in artiodactyl, but the immune events related to protective immunity are not fully understood. To provide a paradigm for a human recombinant antigen vaccine, we have undertaken a vaccination and challenge experiment in pigs, which was recognized as an appropriate animal model in this type of study because of their similarity to human in immunology, and investigated the relative immune events induced by the radiation-attenuated *S. japonicum* cercariae.

**Methods and Findings:**

We found that pigs immunized once with 400 µw UV-irradiated cercariae exhibited 63.84% and 71.82% reductions in worm burden and hepatic eggs respectively. Protective immunity in vaccinated pigs was associated with high level productions of IgM, total IgG, IgG1 and IgG2; IgG2 was significantly increased in the acute infection. IFN-γ levels could be elicited by immunization. At week 6 post-infection, IFN-γ, IL-4 and IL-10 levels also showed a dramatic rise synchronously in vaccinated pigs. Moreover, the *granzyme b*, *nk-lysin*, *ifnγ*, *il4* and *il10* mRNA levels in early skin-draining lymph nodes of immunized pigs were higher than those in pigs with non-irradiated cercariae infection. In addition, cytotoxicity-related genes in the mesenteric lymph nodes were significantly upregulated in vaccinated pigs in the acute infection.

**Conclusion/Significance:**

Our results demonstrated that IFN-γ and IgG2 antibody production, as well as genes related to cytotoxicity are associated with the high level protection induced by UV-irradiated *Schistosoma japonicum* vaccine. These findings indicated that optimal vaccination against *S. japonicum* required the induction of IFN-γ, IgG2 antibody related to Th1 responses and cytotoxicity effect.

## Introduction

Schistosomiasis japonica is still one of the major public health problems that causes significant morbidity and hinders the economic development of endemic areas in China, the Philippines, and Indonesia. In China, schistosomiasis japonica used to be endemic in 12 provinces, and so far seven provinces have still been endemic with an estimated 412,927 cases of schistosomiasis reported in 2008 [1] The control of schistosomiasis japonica is difficult because it is a zoonosis, thus it can be spread through a variety of wild or domestic reservoir hosts, including bovine and porcine [2]. Current measures for schistosomiasis control are based on limited methods such as community chemotherapy, snail control and environmental modifications [3]. In a recent report from a project for a new control strategy, the researchers claimed that a comprehensive control strategy based on interventions to reduce the rate of transmission of *S. japonicum* infection from cattle and human to snail was highly effective in reducing the prevalence and morbidity of the infection [4]. But people still are anxious for the reinfections, which are so common in endemic areas of schistosomiasis japonica and may cripple the disease control efforts. Most studies agree that chemotherapy needs to be complemented with a tool of more long-term effect and an effective vaccine may be an expected choice to provide host with protection against reinfection. Therefore, development of vaccines to protect both humans and domestic animals from the infection or reinfection is still a realistic aim.

In an attempt of developing vaccine against the parasite infection, researchers have devoted a great deal of time and effort in discovering vaccine candidates, most of which have been initially identified in *S. mansoni*, because this species is most easily adapted to laboratory maintenance. Unfortunately, most of these vaccine candidates including DNA vaccines and recombinant vaccines have not been proven to reproducibly provide sufficient immunity in experimental models to warrant consideration for clinical use [5]. The most effective and reproducible protocol to date is vaccination with radiation-attenuated (RA) cercariae, and the vaccine has been identified in *Schistosoma mansoni*. The results of vaccination experiments in a number of animal models (murine, artiodactyl, primates, etc.) in laboratory and field trials have shown that radiation-attenuated vaccines can induce high level, stable protection against challenges with *S. mansoni*. Great progress has been made in the understanding of the immunological mechanisms against *S. mansoni* in mouse models. Both antibody and CD4^+^ T-cell-mediated, IFN-γ-dependent effector mechanisms have been demonstrated using the knockout mice [6], [7], [8]. But the immunologic responses of mice may be marginally relevant to the development of antischistosomal vaccines intended for humans. In contrast, in the case of *S. japonicum*-infected animals, the roles of helper T cell cytokines and antibodies in protective immunity induced by radiation-attenuated cercariae have not been fully understood. Moreover, the majority of studies have shown low level and unstable protection induced by attenuated cercariae in different mouse strains [9], [10], [11], [12], [13], [14]. In domestic animals (artiodactyl), the RA *S. japonicum* vaccine was always able to induce high levels of protection, and the protection exceeded 60% in pig and cattle. But few have involved the possible underlying mechanisms in theses studies except some suggested the involvement of antibody [15], [16], [17], [18], [19]. An understanding of protective human immune mechanisms is essential to engineer an efficacious vaccine formula for human schistosomiasis [5], We need first to understand the immunological responses elicited by RA cercariae, which could pave the way for the human correlated studies. But the existing data from human experiment were too limit to give us a significant help in demonstrating potentially protective immune responses in endemic areas and the immune mechanisms of protection induced by RA vaccine are not easily investigated in humans for ethical reasons [20]. So that it seems very unlikely that in the short term a viable strategy for vaccine development will emerge from studies of infected humans. Obviously, more have to be learned from animal models. Pigs are not only a significant reservoir host of *S. japonicum* in China, but also an animal with close biologic similarities to humans, such as anatomical, physiological as well as immunological properties [14], [21], [22], [23], [24]., Therefore pigs could be a better experimental model to study the relevant protective immune events induced by radiation-attenuated cercariae vaccine. And a comprehensive understanding of the immune parameters in pigs is necessary, which would reflect the protective immune mechanisms in human schistosomiasis.

The purpose of this study is to investigate the correlation of immunological events induced by UV-attenuated cercariae with the protection against challenge infections. We systematically investigated the dynamic characteristics of celluar and humoral immune responses, and explored the relevant molecular events in pigs vaccinated with UV-attenuated cercariae, which could provide a paradigm for the development of optimal vaccine formula for human use.

## Materials and Methods

### Ethics statement

All experiments involving pigs were performed in accordance with protocols approved by the Institutional Animal Care and Use Committee at the Nanjing Medical University (Protocol Number NJMU08-002).

### 1. Animals and parasites

Castrated male Landrace/Yorkshire/Duroc crossbred pigs weighing 18∼20 kg were purchased from Topigs Limit Corporation, China. They were housed together in the farm of the Veterinary Institute of Jiangsu Provincial Agricultural Scientific Academy (Nanjing, China) in controlled sanitary conditions. All pigs were subjected to anti-helminth treatment using albendazole 400 mg twice daily with meals for three days beginning at the 7^th^ day before the experiment. Stool examination showed that all pigs were negative for *S. japonicum*. Sampling procedures and pig sacrifice were performed under intramuscular (i.m.) 1 ml/kg body weight of 3% pentobarbital (except for fecal sampling).

Cercariae were maintained in and released by *Oncomelania hupensis hupensis* hupensis strain, supplied by Jiangsu Institute of Parasitic Disease (Wuxi, China). The snails were collected from Guichi area of Anhui province,China and were infected with the Chinese mainland strain of *S. japonicum* in laboratory of the institute.

### 2. Antigen preparation

*S. japonicum* adult worms were obtained by perfusion of infected rabbits. The adult worms were washed in phosphate-buffered saline (PBS), pH 7.4 containing protease inhibitor cocktail (Sigma), and homogenized several times on ice for 20min. The homogenate was freeze-thawed several times and centrifuged at 30,000 g for 30min at 4°C. The supernatant was passed through a 0.22 µm filter (Millipore Co., USA) and used as soluble adult worm antigen (SWAP).

*S. japonicum* eggs were isolated from infected liver and intestine of rabbit. Purified eggs in PBS with protease inhibitor cocktail were sonicated three times on ice for 10 min. The suspension was freeze-thawed several times and centrifuged at 30,000 g for 30 min at 4°C. The supernatant was passed through a 0.22 µm filter and used as soluble egg antigen (SEA). Protein concentration was determined by BCA protein Assay Kit (Pierce Biotechnology, Inc., IL, USA) [25].

### 3. Immunization schedule

#### 3.1 Preparation of normal cercariae and UV-attenuated cercariae (UVAC)

Briefly, snails were placed in deionized water and exposed to incandescent light for 3 to 4 h. Cercariae were collected from the water surface using a 10 µL bacteriological loop and put on the glass cover slips [26]. Then the cercariae on the cover slip were counted under a dissecting microscope and prepared for infection.

Freshly shed *S. japonicum* cercariae on the cover slip were attenuated with ultraviolet radiation at 254 nm with an intensity of 400 µw/cm^2^ for 1 min using a portable ultraviolet lamp (N16; Konrad Benda, Laborgerate, D-6908 Wiesloch, FRG) as described previously [11], [17]. These attenuated cercariae were used to immunize the animal.

#### 3.2 Vaccination trials

**Figure 1:**
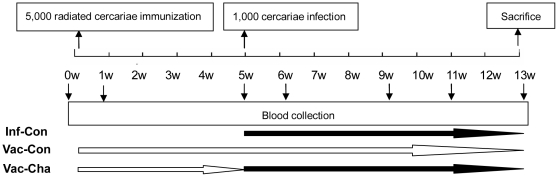
The experiment schedules of immunization, infection and blood collection. White, long arrows represent that the groups were treated with UVAC immunization. Black, long arrows represent that the groups were infected with normal cercariae (*n* = 7).

The pigs were divided into three groups, the vaccination and challenge group (Vac-Cha), the vaccination control group (Vac-Con), and the infection control group (Inf-Con), consisting of seven pigs each. The Vac-Cha and Vac-Con groups were immunized percutaneously with 5,000 UVAC one time. The UVAC were applied to the shaved lateral abdominal flank for 30 min by the cover slip method. At 5 weeks after immunization, the Vac-Cha and Inf-Con groups were infected with 1,000 *S. japonicum* cercariae using the above method. For vaccination and challenge, all experimental pigs were anesthetized with 1 ml/kg body weight of 3% pentobarbital (Figure 1).

### 4. Assessment of vaccine-induced protection

#### 4.1 Adult worm recovery and egg counts in the liver tissue

All pigs were sacrificed at week 8 post-challenge by portal perfusion. Worms were collected and counted from the perfusate sedimentation, and the intestinal mesenteric vessels of each pig were examined for residual worms [27], [28]. A 5-g sample of the left lateral hepatic lobe was digested in 5% KOH for 18 h at 37°C, and five 1-ml subsamples of the digestion fluid were counted for each sample. The mean count was used to determine eggs per gram (EPG) in the liver [29].

#### 4.2 Fecal eggs outputs

The feces collections were performed every two weeks starting 4 weeks post-infection. The filtration and sedimentation Danish Bilharziasis Laboratory (DBL)-technique was used [30]. In brief, a 5-g sample from a homogenized 50∼100 g fecal specimen was mixed with saline, shaken and passed through a series of sieves (400, 100, and 45 µm). The fecal material from the 45 µm sieve was washed into a sedimentation glass, which was filled with saline and left in the dark to sediment. The sediment was centrifuged and resuspended with saline to obtain a volume of 2.25 ml and 150 µl of this solution was mixed with 850 µl 0.9% saline into 1-ml microscope chamber slides. Eggs were counted to obtain the number of eggs per gram (EPG) in the feces.

#### 4.3 Formula of the vaccine-induced protection

Vaccine-induced protection was measured using the percentage of worm or egg reduction in vaccinated groups:

Worm or Egg reduction rate (%) = [numbers of worms or eggs in the Inf-Con group - (numbers of worms or eggs in the Vac-Cha group - numbers of worms or eggs in the Vac-Con group)]/numbers of worms or eggs in the Inf-Con group×100.

#### 4.4 Hepatic histology

Samples from the central part of the left lateral, right lateral, left medial and right medial hepatic lobes were taken from all pigs when they were sacrificed. Then these samples were fixed in 10% buffered formalin and embedded in paraffin. Sections (5 µm) stained with HE (hematoxylin and eosin) were microscopically (Olympus, BX41) examined for histological alterations. 12 slices of four different parts were observed per pigs. Each liver section was examined at 40×magnification to observe the overall inflammation. The distribution of granulomas and cellularity in granulomas formation were observed at 100 or 400×magnification. At the same time, the areas of granulomas containing a single and viable egg were measured and analyzed using a Zeiss microscope (Carl Zeiss, Axiostar, Germany) and KS300 software connected to a Carl Zeiss image analyzer.

### 5. Measurement of specific antibody production

Samples of 5 ml blood were collected from each pig by precaval venipuncture at different time points as shown in Figure 1. The levels of schistosome antigen-specific IgG, IgM, IgG1 and IgG2 antibodies were measured in all swine sera by ELISA technology. Firstly, 96-well microplates (Costar, Roskilde, Denmark) were coated with 100 µl per well of *S. japonicum* soluble egg antigen (SEA, 8 µg/ml for IgG and IgM, 15 µg/ml for IgG1 and IgG2) or soluble worm antigen preparation (SWAP, 15 µg/ml for all four antibodies) at 4°C overnight. The plates were washed once with 0.05% PBS-Tween 20 (PBST). After adding 200 µl blocking buffer (5% skimmed milk), the plates were incubated at 37°C for 1 h and then washed with PBST. Secondly, after two washes with PBST, each serum was diluted 1∶100 with PBS, and added to each well. The plates were incubated for 2 h at 37°C. Thirdly, after five additional washes with PBST, HRP-conjugated goat anti-pig IgG (Serotec, AHP865P) or goat anti-pig IgM (Serotec, AAI39P) was diluted 1∶30,000 with PBS, and 100 µl was added to each well, then incubated at 37°C for 1 h. For IgG1 or IgG2 detection, the wells were incubated with 100 µl per well of 1/100-diluted mouse anti-porcine IgG1 (Serotec, MCA635) or IgG2 (Serotec, MCA636) at 37°C for 1 h, respectively. After washes, the wells were incubated at 37°C for 1 h with 100 µl per well of 1/10,000-diluted HRP-conjugated goat anti-mouse IgG. Fourthly, after five washes with PBST, the plates were developed with TMB substrate (AMERESO) for 30 min and the reaction was stopped by adding 50 µl H_2_SO_4_ (2 M). The plates were read at 450 nm, using an ELISA reader (Bio-Rad mod. 550). Each serum sample was tested in triplicate. A serum sample from a pig with acute *S. japonicum* infection served as a positive control, and a sample from an uninfected pig was used as a negative control.

### 6. Measurement of cytokine production

#### 6.1 Peripheral blood mononuclear cells (PBMC) preparation

The 20 ml blood samples were collected in heparinized tubes by precaval venipuncture at different time points as shown in Figure 1. PBMC were isolated using swine lymphocyte separation medium (density: 1.110, Haoyang Bioproduction Limit Corporation, Tianjin, China). PBMC were cultured at 1.5×10^6^/ml in RPMI 1640 medium supplemented with penicillin (100 U/ml), streptomycin (100 µg/ml) and 10% fetal calf serum in 96-well flat-bottomed microtiter plates (Costar, Roskilde, Denmark) in the presence of either 12.5 µg/ml phytohemagglutinin (PHA), 50 µg/ml SEA, or 50 µg/ml SWAP, or in complete RPMI 1640 alone in triplicate. PBMC were incubated at 37°C in a humidified atmosphere with 5% CO_2_ for 72 h and the supernatants were harvested for cytokine determination.

#### 6.2 Determination of cytokine levels

IFN-γ, IL-4 and IL-10 were measured using the swine ELISA Kits following the manufacturer's instructions (Invitrogen Corporation, California, USA). The plates were read at 450 nm using an ELISA reader, and the levels of IFN-γ, IL-4 and IL-10 in the examined supernatants were determined using a standard curve constructed with cytokine standards provided by the manufacturer.

### 7. Genechip analysis and semi-quantitative RT-PCR

#### 7.1 Isolation of skin-draining lymph nodes (sdLNs) and mesenteric lymph nodes

The skin-draining lymph nodes (inguinal lymph nodes) from three pigs in the Vac-Con group were isolated on day 7 after immunization, and sdLNs in the Vac-Cha and Inf-Con groups were isolated on day 7 after infection. All the sdLNs were immediately put into liquid nitrogen for storage. When the pigs were sacrificed at week 8 post-challenge, the mesenteric lymph nodes surrounding the cecum from three pigs in each of the Vac-Con, Vac-Cha and Inf-Con groups were isolated immediately and also stored in liquid nitrogen.

#### 7.2 Microarray hybridization and analysis

The affymatrix porcine genome arrays were analyzed in the Shanghai Biochip Co. Ltd. Total RNA extraction from each mesenteric lymph node was performed with TRIzol reagent (Invitrogen Life Technologies) and purified with RNeasy mini kits (QIAGEN). Equal amounts of total RNA from 3 pigs per group were mixed, cDNA was generated using One-Cycle Target Labeling and Control Reagents (Affymetrix), and cRNA was made with GeneChip® IVT Labeling Kit (Affymetrix). Biotin-labeled, fragmented (200 nt or less) cRNA was hybridized for 16 h at 45°C to Affymetrix porcine genome arrays by the Shanghai Biochip Co.,Ltd. The arrays were washed, stained, and read with a GeneChip® Scanner 3,000. The fluorescence signal was excited at 570 nm, and data were collected on a confocal scanner at 3-µm resolution. Data sorting and analyses were performed using GeneSpring GCOS1.4 software. All datas were MIAME compliant and the raw datas had been deposited in a MIAME compliant database (GEO accession number was GSE22311).

#### 7.3 Quantitative RT-PCR

Total RNA of each sdLNs and mesenteric lymph node was extracted using TRIzol reagent (Invitrogen, Life Technologies), purified with the RNeasy mini kit (QIAGEN), and reverse-transcribed to cDNA using the PrimeScript RT reagent kit (Takara). cDNA was amplified with SYBR Green Master (Roche) in a 7300 Real-time PCR System (Applied Biosystems). Primers for qRT-PCR were designed using Real-time PCR Primer Design (Table 1). Real-time PCR was run in triplicates in a volume of 20 µl containing 10 µl of SYBR Green PCR Master, 300 nM of each primer, and 50 ng cDNA. Reaction conditions were as described for the SYBR Green kit and the cycling protocol was as follows: 50°C for 2 min, 95°C for 10 min, 40 cycles of 95°C for 15 s, 60°C for 1 min. The housekeeping gene *β-actin* was used as an internal control. Quantitation of relative differences in expression were finally calculated using the comparative 2^−ΔΔCT^ method [31].

**Table 1:** Primers, annealing temperatures and number of cycles used for the amplification of each target gene.

Target gene	Oligonucleotide primers	Annealing temperature (°C)	Number of cycles	Product size (bp)
*beta-actin*	F: 5′CCAAAGCCAACCGTGAGA 3′R: 5′ CCAGAGGCGTACAGGGACA3′	50	40	103
*granzyme b*	F: 5′ AAGTTGGTGCGGTGGGTT3′R: 5′ AGGGTTATAGTCTGGGTGATGG3′	50	40	169
*nk-lysin*	F: 5′ GCCTCATCTGTGAGTCTTGTCG3′R: 5′ CAGTGTCCTCGTTGGGTTGTG3′	50	40	78
*tnfα*	F: 5′TGGCCCCTTGAGCATCA AC3′R: 5′CGACGGGCTTATCTGAGGTTT3′	50	40	71
*ifnγ*	F: 5′ TGGTAGCTCTGGGAAACTGAATG3′R: 5′ TGGCTTTGCGCTGGATCTG3′	50	40	80
*Il12p40*	F: 5′ GATGCTGGCCAGTACACC3′R: 5′ TCCAGCACGACCTCAATG3′	50	40	111
*Il4*	F: 5′ GGACACAAGTGCGACATCA3′R: 5′ GCACGTGTGGTGTCTGTA3′	50	40	186
*Il10*	F: 5′ CGGCGCTGTCATCAATTTCTG3′R: 5′ CCCCTCTCTTGGAGCTTGCTA3′	50	40	89
*cxcl12*	F: 5′ TACAGATGCCCTTGCCGATT3′R: 5′ TGTTGCTCTTCAGCCGTGC3′	58	40	118
*igfbp3*	F: 5′ CGAGGAGGACCGCAGTGTA3′R: 5′ TGGCGTGCCCTTTCTTGAT3′	58	40	122

### 8. Statistical analysis

All statistical analyses were performed using SPSS for Windows 13.0. The data were expressed as mean ± SEM. The effect of treatment and time was examined for significance using the two-way ANOVA test. One-way ANOVA was performed to test for differences in groups at the same time point. For all tests, a *P* value less than 0.05 denoted statistical significance.

## Results

### 1. Statistical analysis of the effects of treatment and time in the experiment

The number of eggs in feces and some of immune measurements are defined by two parameters, treatment and time. The two-way ANOVA test was performed to assess these data. The results showed the significant effects of time and treatment, and there was a significant interaction between these two parameters (*P*<0.05). The *P* values were showed in table 2.

**Table 2:** *P* values obtained from the statistical analysis of the effects of the treatment and time in the experiment.

Parameters		Treatment	Time	Time* treatment
Fecal egg output		2.672279169477e-016	2.14499577444e-018	1.296002073369e-012
Antibody level	SWAP specific IgM level	6.682219419948e-007	4.04666814603e-042	4.158940098223e-007
	SEA specific IgM level	5.572100354147e-014	9.641207055032e-043	2.407000312568e-027
	SWAP specific IgG level	5.52128827454e-029	2.451622481746e-051	1.376649356814e-031
	SEA specific IgG level	1.053306125485e-025	3.699534157222e-063	1.769465429139e-039
	SWAP specific IgG1 level	9.228280791802e-014	9.054759310497e-037	3.449782822025e-017
	SEA specific IgG1 level	1.868026578109e-015	4.16012333539e-043	2.15332915489e-028
	SWAP specific IgG2 level	4.7543256879e-009	3.620423974106e-021	1.458097936007e-007
	SEA specific IgG2 level	6.300423181744e-008	9.366827607566e-014	2.871458659388e-008
Cytokine level	IFN-γ levels stimulated with SWAP	4.490487936647e-008	3.389161767425e-011	1.730663325338e-015
	IFN-γ levels stimulated with SEA	2.166712830768e-020	3.148512447309e-024	6.329193456019e-036
	IL-10 levels stimulated with SWAP	0.000175321646018	9.59486120791e-005	3.744079791233e-009
	IL-10 levels stimulated with SEA	1.365638359872e-007	3.492905669287e-017	1.093071684209e-024
	IL-4 levels stimulated with SWAP	8.809962570855e-005	6.602527317794e-009	7.247692710049e-010
	IL-4 levels stimulated with SEA	0.0102200467708	1.787895972731e-009	2.07591663806e-006

### 2. UVAC immunization induced high protection against *Schistosoma japonicum*

#### 2.1 Reduced parasite burden in UVAC-vaccinated pigs

The number of adult worms recovered and hepatic eggs per gram in Vac-Cha group was significantly lower than that in Inf-Con group at week 8 post-challenge (*P*<0.01) as shown in.table 3. UVAC vaccination (Vac-Cha group) led to a 63.84% reduction in number of worms and 71.82% hepatic egg burden compared to the Inf-Con group. The reduction in egg number was roughly consistent with that of female adult worms. The number of female adult worms in the Vac-Cha group was reduced by 67.65% (*P*<0.01). Thus, UVAC could induce a high level of protection against a *S. japonicum* challenge.

**Table 3:** Comparison of adult worms and eggs per gram liver tissue among three groups of pigs.

Group	Number of pig	Average number of worms ( 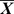 ±SEM)	Average number of female worms ( 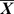 ±SEM)	Average number of eggs pergram liver tissue ( 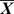 ±SEM)
Vac-Cha	7	118.71±16.31**(63.84%)†	53.42±6.62**(67.65%)†	74.86±7.34**(71.82%)†
Inf-Con	7	328.29±41.33	165.14±20.64	262.57±51.60
Vac-Con	7	0	0	0

**P<0.01, compared with the Inf-Con group.

† represents worm reduction rate (%) or egg reduction rate (%) in liver tissue.

#### 2.2 Less fecal egg output in UVAC-vaccinated pigs

**Figure 2:**
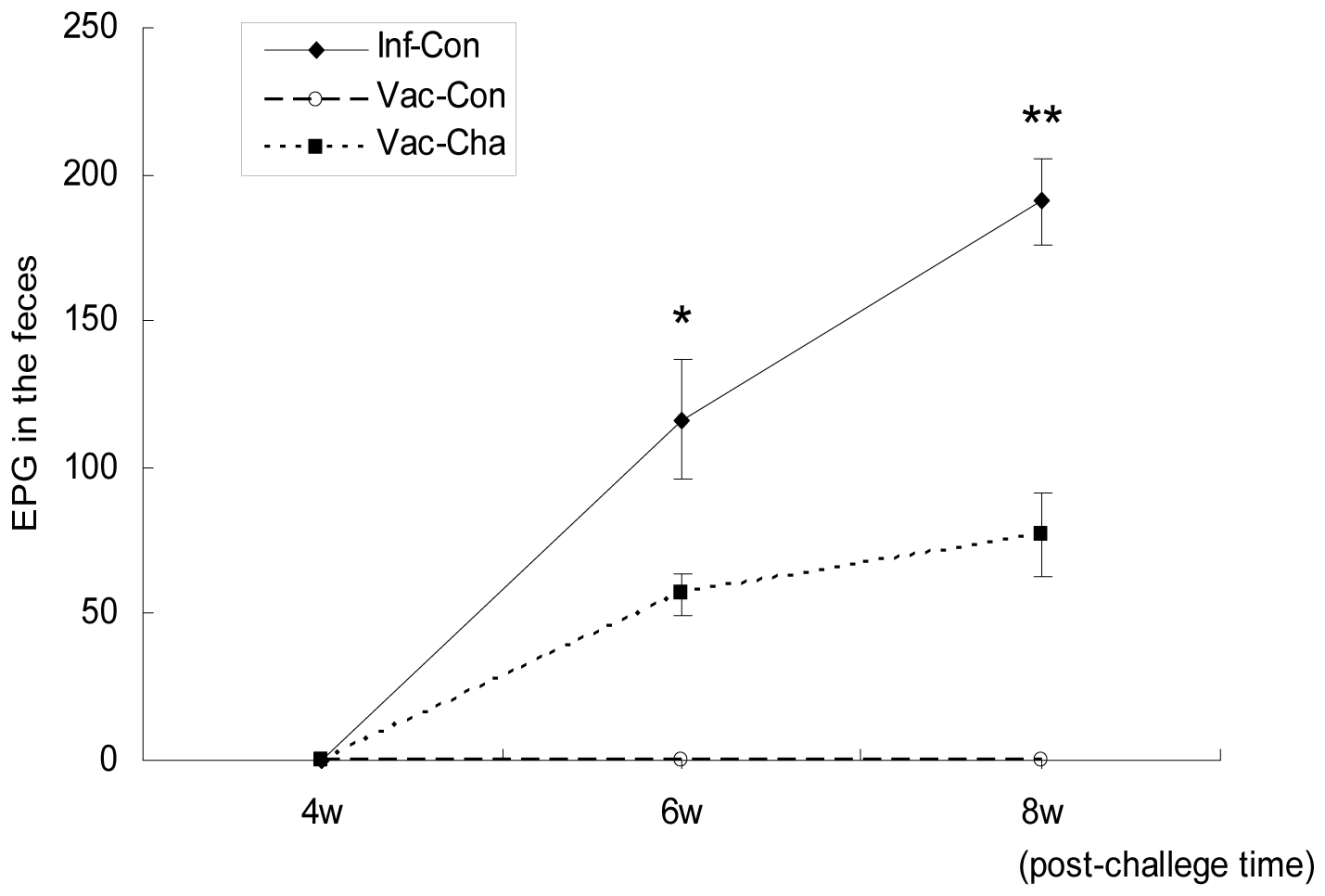
The dynamic change of fecal egg output after *Schistosoma japonicum* infection. The numbers of EPG in feces at weeks 4, 6 and 8 post-infection are shown. *P value<0.05 and **P value <0.01 compared to the Vac-Cha group (*n* = 7).

In order to monitor the dynamic changes of infection and estimate the parasite burden in the body, fecal samples were collected to analyze egg numbers starting at 4 weeks post-infection. The level of fecal egg output in the UVAC vaccination group was significantly lower than that in the Inf-Con group at 6 weeks post-infection. At week 8 after *S. japonicum* challenge, the reduction of Vac-Cha group to Inf-Con group in the number of fecal eggs was 76.76%. No fecal eggs were found in the Vac-Con group (Figure 2).

#### 2.3 Alleviated liver histopathology in UVAC-vaccinated pigs

**Figure 3:**
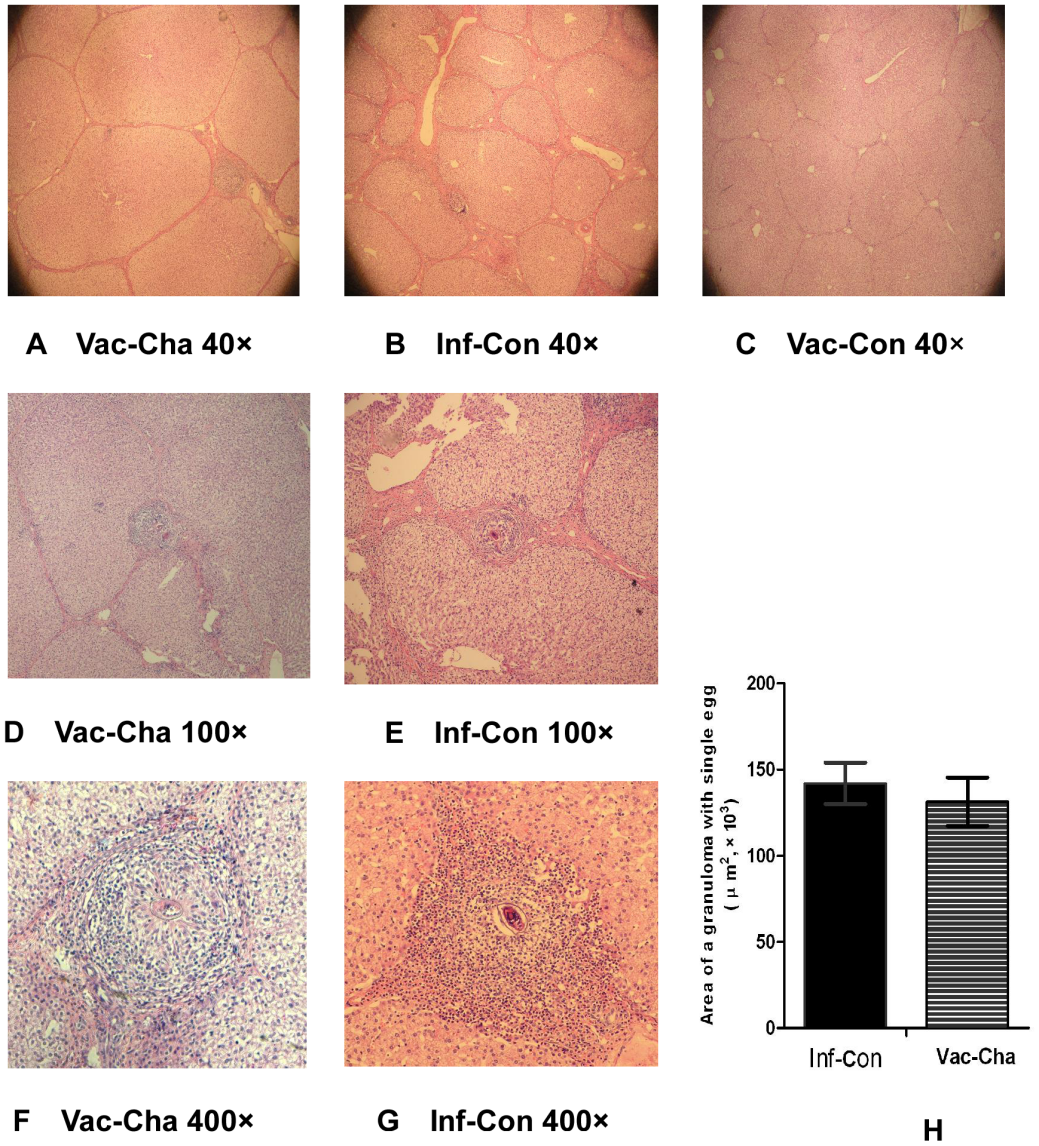
Liver histology at 8 weeks after infection with HE staining and the average area of a single granuloma containing one egg. Figures 3A, 3D and 3F show fewer granulomas and mild inflammation in the livers of the Vac-Cha group. Figures 3B, 3E and 3G show severe granulomatous inflammation in the livers of the Inf-Con group. Figure 3C shows no granuloma in the livers of the Vac-Con group. Figure 3H shows the average area of a single liver granuloma containing only one egg in the Vac-Cha and Inf-Con groups (*n* = 7).

Figure 3 shows the liver histology of the three groups at week 8 after infection. The liver sections from the Vac-Con group showed no granulomas and little inflammation (Figure 3C), whereas inflammatory reaction was observed in the liver samples of the Vac-Cha and Inf-Con groups (Figures 3A and 3B). In the Vac-Cha group, small and diffuse granulomas occurred, and mild inflammation was observed in the livers (Figures 3D and 3F). Bigger and more numerous granulomas and severe inflammation were observed in the Inf-Con group (Figures 3E and 3G). All numbers and mean areas of single-egg granulomas were observed in 12 slices per pigs of different groups. Although the mean areas of single-egg granulomas showed little difference between the Inf-Con and Vac-Cha groups, 57 granulomas containing a single egg were found in the Inf-Con group and only 30 were found in the Vac-Cha group (Figure 3H).

### 3. UVAC immunization enhanced the antibody response to *Schistosoma japonicum* infection

#### 3.1 Elevated IgM and IgG antibody response from immunization to early stage of infection in UVAC-vaccinated pigs

**Figure 4:**
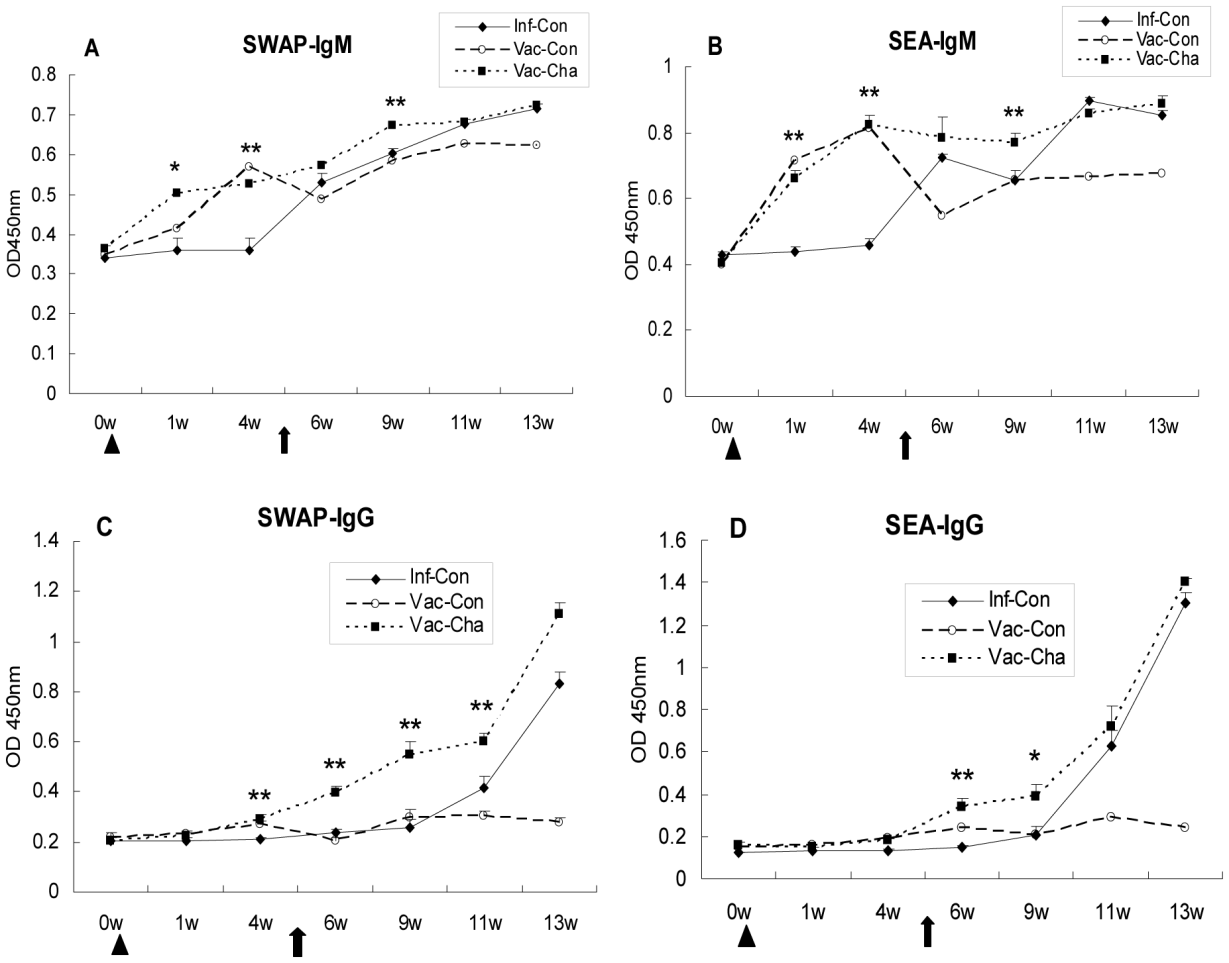
Specific IgM and IgG responses to SWAP and SEA. The Vac-Cha group was immunized with UVAC at week 0 and challenged with normal cercariae at week 5 as well as the Inf-Con group. Triangles and arrows show the times of immunization and infection, respectively. *P<0.05 and **P<0.01 compared to the Inf-Con group. Data are representative of two independent experiments with similar results (*n* = 7).

In this experiment, IgM responses to SWAP and SEA in the Vac-Cha group arose immediately after immunization and early infection, earlier than those for IgG antibody. The IgM levels in the Vac-Cha group were higher than those in the Inf-Con group. The differences between the Vac-Cha and Inf-Con groups were significant at weeks 1, 4, and weeks 4 post-challenge (*P*<0.01) as shown in Figure 4. Until the acute stage of infection, IgM expression in the Vac-Cha and Inf-Con groups showed little difference.

The dynamic IgG response patterns to two schistosome-specific antigens were similar. In the Vac-Cha group, the SWAP-specific IgG levels began to rise at 2 weeks after immunization and generally exceeded those in the Inf-Con group, especially in the course of immunization and early stage of infection. The increase of SEA-specific IgG occurred later than that for SWAP-specific IgG levels, and the trend was similar for the Vac-Cha and Inf-Con groups after the onset of a large number of eggs. The IgM and IgG levels of the Vac-Con group increased after immunization and reached a peak at week 4, then decreased gradually.

#### 3.2 Significant enhancement of IgG2 expression in the acute infection in UVAC-vaccinated pigs

**Figure 5:**
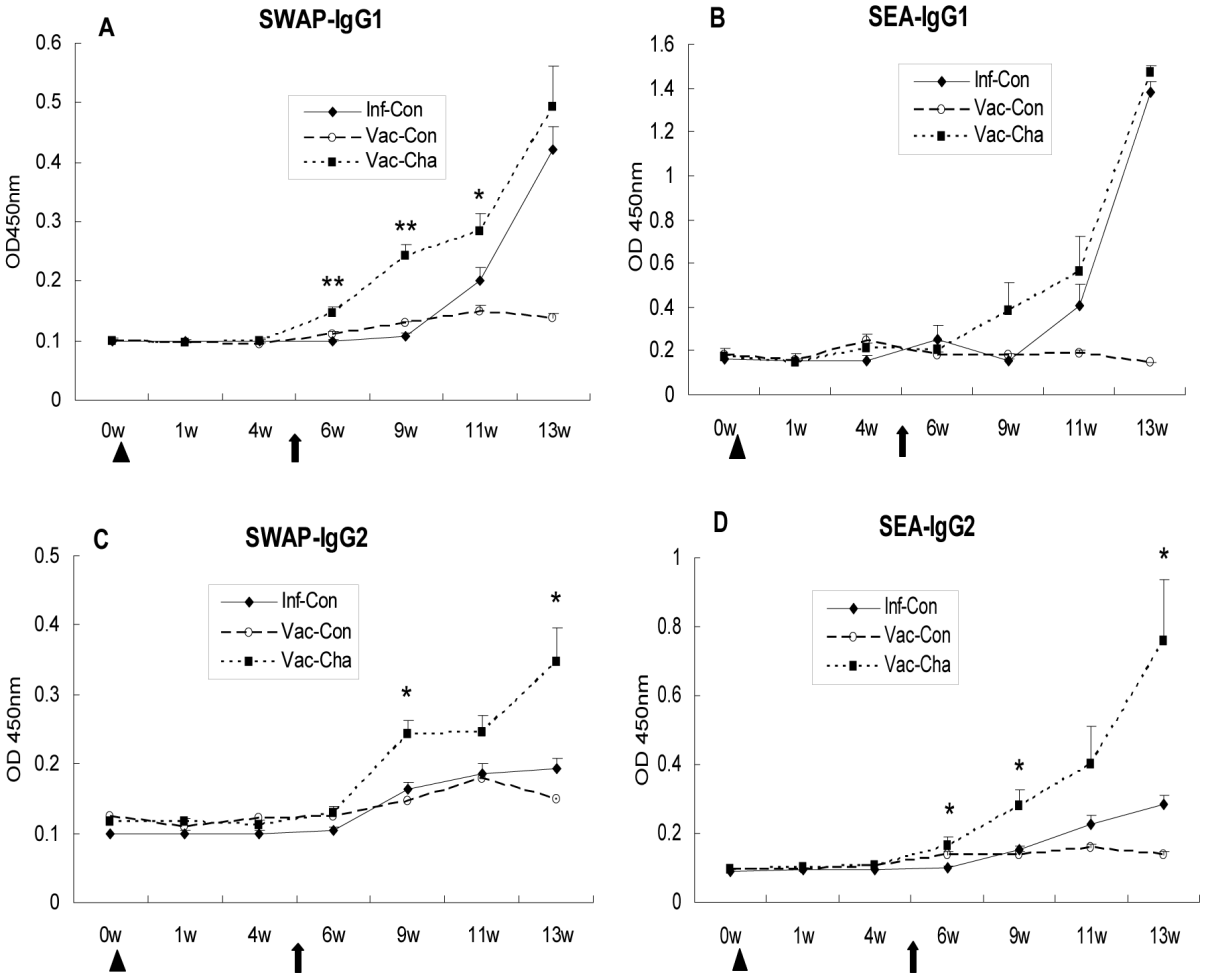
Specific IgG1 and IgG2 responses to SWAP and SEA. All groups were treated as described for Figure 4. *P<0.05 and **P<0.01 compared to the Inf-Con group. Data are representative of two independent experiments with similar results (*n* = 7).

The expression pattern of IgG1 subtype antibody was very similar to that of total IgG antibody. Anti-SWAP IgG1 levels in the Vac-Cha group were significantly higher than those in the Inf-Con group at weeks 1, 4, and 6 post-challenge. Anti-SEA IgG1 levels in the Vac-Cha group began to increase at week 4 post-challenge, but there was no statistically significant difference compared with those in the Inf-Con group. However, the levels of IgG2 in pigs with UVAC vaccination were significantly higher than those in the Inf-Con group after *S. japonicum* infection. Anti-SWAP IgG2 levels were significantly increased at weeks 4 and 8 post-challenge, and anti-SEA IgG2 levels were higher at weeks 1, 4, and 8 post-challenge (*P*<0.05) in the Vac-Cha group than in the Inf-Con group (Figure 5). The Vac-Cha and Vac-Con groups had lower SWAP-specific or SEA-specific IgG1/IgG2 ratios than the Inf-Con group at week 13 (Table 4). The IgG1 and IgG2 levels in the Vac-Con group were low after infection.

**Table 4:** Comparison of IgG1/IgG2 ratio in the acute infection with *Schistosoma japonicum* among the three groups.

week	SWAP-specific IgG1/IgG2			SEA-specific IgG1/IgG2		
	Inf-Con	Vac-Con	Vac-Cha	Inf-Con	Vac-Con	Vac-Cha
11w	1.78	1.21	1.4	1.09	0.83	1.05
13w	4.87	1.03	1.93	2.19	0.93	1.43

### 4. UVAC vaccination elicited a mixed Th1/Th2 phenotype in the acute infection, and a significant increase in IFN-γ production after immunization

**Figure 6:**
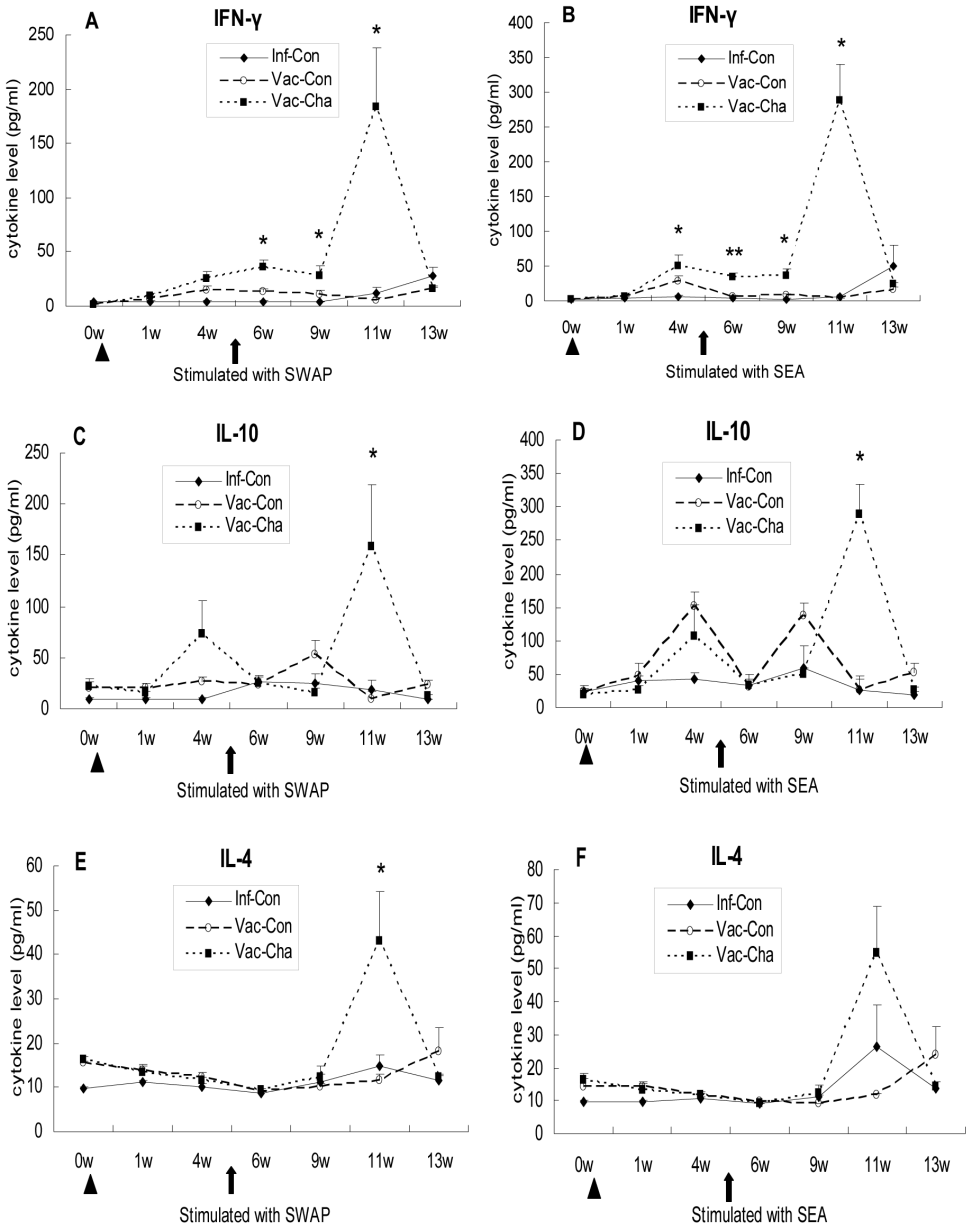
IFN-γ, IL-10 and IL-4 produced by PBMC stimulated with SWAP and SEA. Triangles and arrows show the times of immunization and infection, respectively. *P<0.05 and **P<0.01 for the Vac-Cha group compared to the Inf-Con group. Data are representative of two independent experiments with similar results (*n* = 7).

In order to further investigate the immunological mechanism of the high protection in UVAC-vaccinated pigs, the levels of IFN-γ, IL-10 and IL-4 in the PBMC culture supernatants were quantified by ELISA (Figure 6). Firstly, in the Vac-Cha group, IFN-γ concentrations in response to SWAP and SEA began to increase after immunization and were significantly higher than those in the Inf-Con group; in particular, IFN-γ had a dramatic rise at week 6 post-challenge. Secondly, IL-10 levels in the Vac-Cha group had an increment after immunization and increased significantly at week 6 post-challenge in response to SWAP and SEA. IL-10 expression in the Inf-Con group showed relatively low levels compared with those in the Vac-Cha group. Finally, the level of IL-4 in the Vac-Cha group only increased at week 6 post-challenge, and levels of this cytokine in the other groups remained low at all times. In the Vac-Con group, IFN-γ and IL-10 expression increased initially after immunization and then decreased. IL-4 concentrations were low throughout the experiment. The PBMC stimulated with PHA produced the highest levels of the three cytokines, while blanks without any antigen stimulation produced low levels in all groups for both experiments (data not shown). Two independent experiments on pigs showed similar results.

### 5. UVAC vaccination stimulated transcription of genes related to early CD4 and CD8 responses in sdLNs (inguinal lymph nodes)

**Figure 7:**
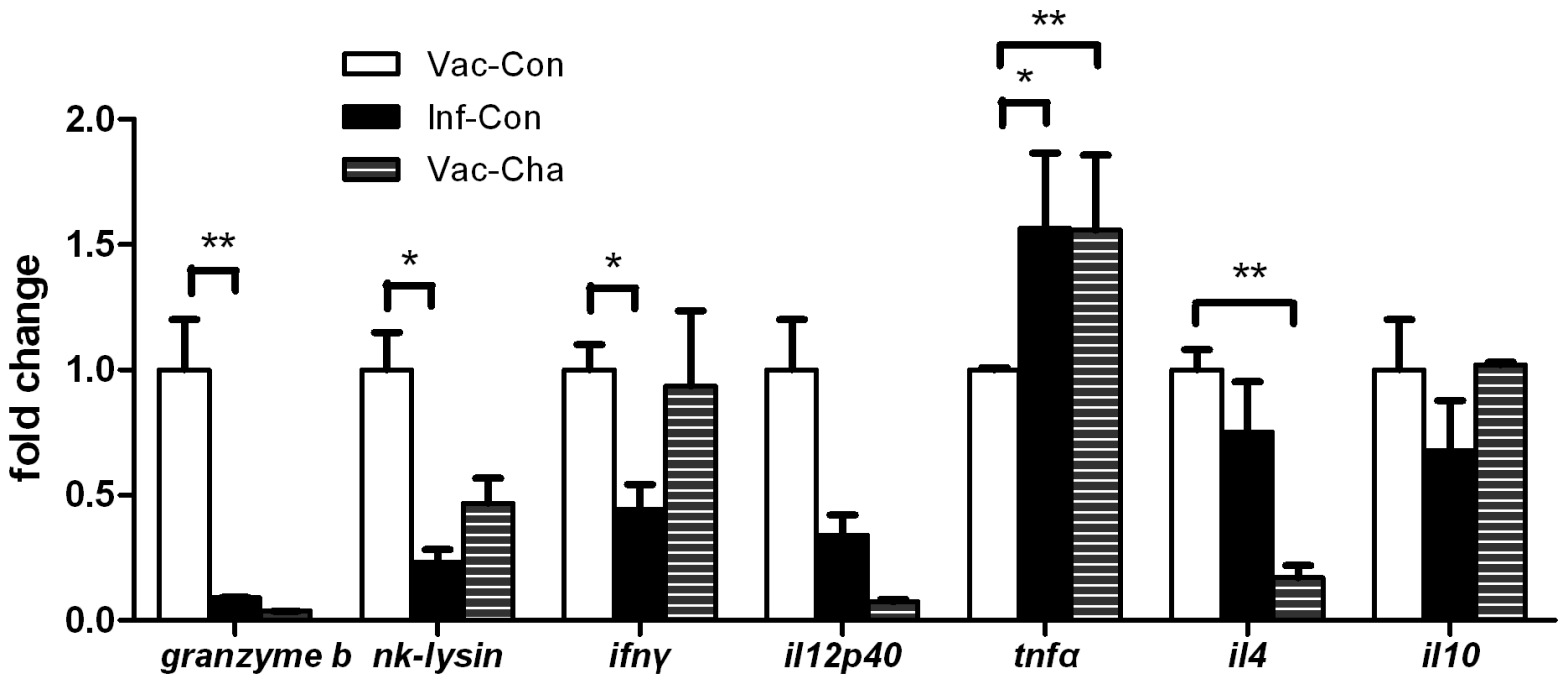
The relative mRNA levels of *granzyme b*, *nk-lysin*, *ifn γ*, *il12p40*, *tnf α*, *il4* and *il10* in sdLNs. The sdLNs were isolated from three pigs in the Vac-Con group on day 7 after attenuated cercariae immunization, and sdLNs in the Vac-Cha and Inf-Con groups were isolated on day 7 after normal cercariae infection, respectively. *P<0.05 and **P<0.01 (*n* = 3).

To observe differences in early CD4^+^ T and CD8^+^ T functions in sdLNs following percutaneous exposure to attenuated or normal cercariae, transcription of certain genes in sdLNs was tested by quantitative RT-PCR on day 7 after immunization or infection. Seven days after immunization with UV-attenuated cercariae, the *granzyme b*, *nk-lysin*, and *ifnγ* mRNA levels in sdLNs were significantly higher than those in sdLNs infected with normal cercariae. Although *il12p40*, *il4*, and *il10* levels after immunization were increased compared with post-infection, the differences were not significant. The *tnfα* level after immunization was markedly lower than that after infection (Figure 7).

### 6. Cytotoxicity-Related Genes were markedly upregulated in the mesenteric lymph nodes of UVAC-vaccinated pigs

**Figure 8:**
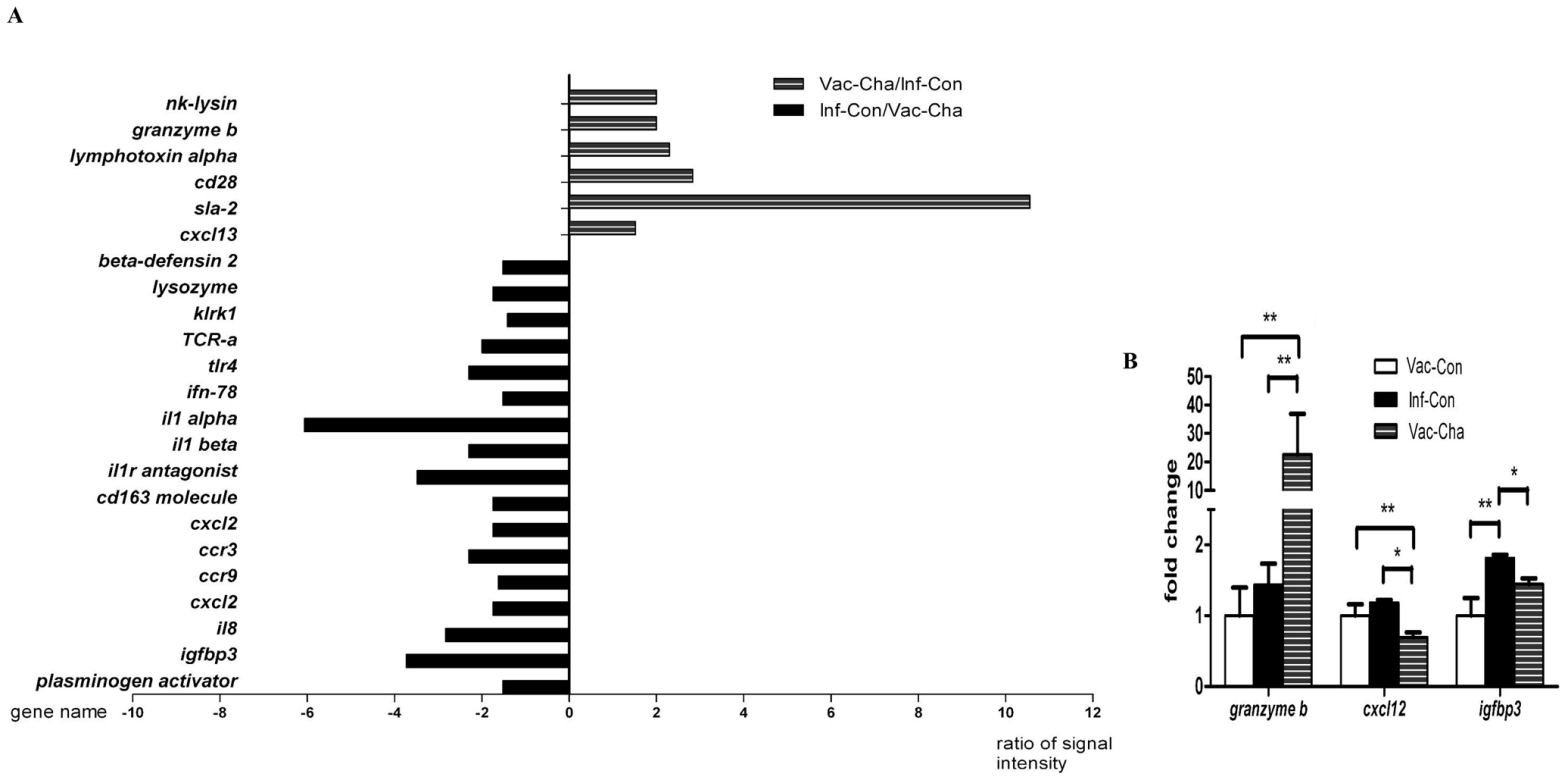
Relative mRNA signal intensities for certain genes of mesenteric lymph nodes in the Vac-Cha and Inf-Con groups assayed by microarrays and validated by qRT-PCR. In figure 8A, the numbers along the X-axis represent the ratio of signal intensities between the Vac-Cha and Inf-Con groups. The positive values on the right-side X-axis: gene signal intensity of the Vac-Cha group/gene signal intensity of the Inf-Con group; the negative values of the left-side X-axis: gene signal intensity of the Inf-Con group/gene signal intensity of the Vac-Cha group. The gene names are listed along the Y-axis. Figure 8B shows the relative mRNA levels of *granzyme b*, *cxcl12*, and *igfbp3* for the Vac-Con, Inf-Con and Vac-Cha groups. *P<0.05 and **P<0.01 (*n* = 3).

The mesenteric lymph nodes surrounding the cecum from the Vac-Cha and Inf-Con groups were collected at week 8 post challenge and used to analyze the gene profile with Affymetrix oligo-probe arrays. In the Vac-Cha group, signal intensities of 185 genes were significantly upregulated compared to the Inf-Con group,with a fold change of 1.5 or greater. Among them were cytotoxic effect genes (*lymphotoxin alpha*, *granzyme b*), porcine host defense peptide gene (*nk-lysin*), cell surface markers associated with the activation of T cells (Leukocyte mRNA for MHC II antigen, *sla-2*, *cd28 antigen*, TCR alpha chain mRNA C-region) and B cell chemokine gene (*cxcl13*). However, the signal intensities of 207 genes were significantly higher in the Inf-Con group than those in the Vac-Cha group. Some of these fold changes may reflect the higher parasite burden and helminth infection-associated immune responses of the Inf-Con group. One of the most prominent groups of stimulated genes were those coding for inflammation-response factors, chemokine genes and chemokine receptor genes, including *cd163*, *il1r antagonist*, *il1 beta*, *il1 alpha*, *ifn-78k*, *cxcl12*, *ccr9*, *ccr3*, *cxcl2*, *il8*, *killer cell lectin-like receptor subfamily k*, *tlr4*, *insulin-like growth factor binding protein 3* (*igfbp3*), IFN alpha-inducible protein, inflammatory response protein 6, eosinophil chemotactic cytokine, and macrophage receptor with collagenous structure. Some representative gene signal intensities are shown in Figure 8A.

To validate the differential expression of the genes identified by the microarrays, quantitative RT-PCR was used to detect some representative gene expression levels in the mesenteric lymph nodes from pigs of the Inf-Con and Vac-Cha groups at week 8 post-challenge. The *granzyme b* mRNA level in the Vac-Cha group increased significantly, compared with the Vac-Con and Inf-Con groups, while the expression of *cxcl12* and *igfbp3* in the Inf-Con group were significantly higher than those in the Vac-Cha group (Figure 8B).

## Discussion

The experiments described here have ascertained that Landrace/Yorkshire/Duroc crossbred pigs immunized by 400 µw UV-irradiated *S. japonicum* cercariae exhibited high levels of protection against a challenge with *S. japonicum*. Immunization resulted in significant decreases in worm number in portal hepatic and intestinal mesenteric systems, and egg numbers in feces and liver, as well as alleviated liver pathology compared with infected pigs without pre-vaccination. These results are consistent with our previous work in that the worm reduction exceeded 60% in pigs vaccinated with UV-irradiated *S. japonicum* cercariae. In other laboratories, the protection against *S. japonicum* in pigs could reach 56%∼95% worm reduction with ultraviolet-attenuated cercariae vaccination, or about 95% worm reduction with γ-attenuated cercariae immunization [16], [18], [19]. Our results added support to our hypothesis that radiation-attenuated cercariae (RA) could induce high protection against *S. japonicum* infection in big animals.

Immunological parameters were monitored throughout the experiment in order to correlate the protection with elicited immune responses, leading to better understanding of the protective mechanism induced by UV-attenuated cercariae. The results show that the high level protection induced by UV-attenuated cercariae in pigs was associated with high IFN-γ levels produced by cell-mediated responses, and IgG2 levels produced by humoral immune responses. Firstly, the results of PBMC culture stimulated with SWAP and SEA *in vitro* showed that the IFN-γ levels could be elicited by UVAC immunization. Then, challenge of the vaccinated pigs with normal cercariae could further increase the IFN-γ production in the PBMC culture stimulated with SWAP, possibly associated with the reaction of memory T cell. Meanwhile, we also observed higher IL-10 production after immunization, which could enable down-regulation of the type 2 response [32]. But IL-4 was not induced by immunization with the lowest level at week 1 post-challenge in the Vac-Cha group. So IFN-γ is likely crucial for vaccine-induced protection after immunization in the pig. This is consistent with previous data obtained with large mammals. Exposure of chimpanzees to RA vaccine also induced a type 1 cytokine response after the vaccination, as measured by the rapid increase in IFN-γ in SWAP- and SEA-stimulated PBMC cultures [32].The level of IFN-γ in the RA-immunized Grivet monkeys was higher than in controls [33].The involvement of IFN-γ in protective immunity to schistosomiasis is well-documented for the murine model. In addition, after attenuated cercariae vaccination, a proportion of schistosome-specific Th1 cells generated in the sdLNs are likely to become memory cells, and could promote the production of large amounts of cytokines and provoke delayed type hypersensitivity (DTH) reactions following secondary schistosome challenge [34], [35]. At the acute stage of infection, besides IFN-γ, the expression of IL-4 and IL-10 in the Vac-Cha group increased significantly at week 6 post-challenge. The adaptive immune response in single vaccination animals was dominated by a mixed Th1/Th2 phenotype [32], [33]. The regulatory cytokine IL-10 might contribute to prevent the development of highly polarized Th1 or Th2 responses and alleviate liver pathology. Other cytokines, such as transforming growth factor β, or cellular processes, such as apoptosis, might be involved [32]. Therefore, these results suggested that the ideal vaccine would need to induce IFN-γ especially at the early stage of immunization but avoid over-induction of down-regulatory cytokines. Secondly, based on irradiated cercariae studies in various host species, immunizing regimens that are believed to induce antibody-mediated immunity have been shown to lead to long-lived protection. Our results also showed that the IgM level rapidly increased after immunization, and IgG, IgG1 and IgG2 levels in Vac-Cha group did not rise significantly until challenge with normal cercariae.. However only the IgG2 expression in Vac-Cha group was significantly higher than those in Inf-Con group at the acute stage of infection. In pigs, IFN-γ and IL-12 induced a bias towards IgG2 production; since IL-10 could up-regulate IgG1 levels, the IgG2 subclass is considered to be associated with a Th1-cell-controlled immune response, while the IgG1 subclass is associated with a Th2-cell-controlled response [36]. In the Vac-Cha group, the high level of IFN-γ at week 6 post-challenge promoted switching to IgG2, and the degree may be stronger than the IL-10 regulation on IgG1. In humans infected with *S. mansoni*, the high levels of SWAP-specific IgG2 were associated with lower infection levels, and IFN-γ in humans also stimulated the production of IgG2 [37]. Homologous passive transfer of protection using sera from singly or multiply vaccinated rats indicated that the IgG2a antibody is the principal mediator of protection in these animals. And in rats, complement depletion during passive transfer at the lung stage significantly reduced protection, thus generation of C3a and C5a by antibody/antigen fixation of complement may play a role [7]. Consistently, porcine IgG2 is more effective than IgG1 for activation of complement [38]. Therefore we conclude that the Th1-prone IgG2 subclass is more indicative of protective efficiency against *S. japonicum* in the acute infection stage. So the IgG2 antibody -mediated immunity may promote long-lived protection and is important for the schistosomiasis vaccine development.

In addition to the effect of IFN-γ and IgG2, the activation of cytotoxic genes might contribute to high protection at the early stage of UVAC immunization and acute stage of infection. Our results showed that after attenuated cercariae immunization, *granzyme b*, *nk-lysin*, *ifnγ*, *il12p40*, *il4* and *il10* genes expression levels, related with CD8^+^T and CD4^+^ T, were higher than that after normal cercaria infection. In mouse and pig models, the T cell and DC proliferation in sdLN induced by irradiated cercariae is stronger than that induced by normal cercariae, and RA vaccination preferentially induces the accumulation of IFN-γ in the sdLN of mice [39], [40], [41]. Fragile bodies and slow migration in the RA could release a great amount of parasite antigens, or prolong the exposure to the host immune system, which may in turn favor the priming of protective responses [7]. Our results implied that the function of T cells in sdLN induced by irradiated cercariae was enhanced than induced by normal cercariae. In addition, at the acute stage of infection, the analysis of gene expression profiles of the mesenteric lymph nodes from the Vac-Cha and Inf-Con groups, which best represent the immune response to eggs in schistosome infections [42], support the notion that the killer cell-mediated cytotoxicity might have played an important part in immune protection in the Vac-Cha group, whereas in the Inf-Con group, the hosts mounted typical immune responses against helminth infection. Some significantly differentially expressed genes, such as cytotoxic effector genes, *granzyme b* and *nk-lysin*, showed stronger signal intensities in the Vac-Cha group compared to the Inf-Con group. Granzymes are a family of highly homologous serine proteases that are released by cytoplasmic granules within cytotoxic T cells (CTL) and/or natural killer cells (NK). It is well-accepted that perforin helps granzymes to enter cells by various pathways, causing apoptosis [43]. NK-lysin is one kind of host defense peptide (HDP) produced by pig cytolytic lymphocytes. HDPs, usually termed antimicrobial peptides, are important components of innate immunity and provide primary immune protection [44]. Thus, the enhanced expression of cytotoxicity-related genes in the low parasite burden Vac-Cha group suggests that killer cell-mediated cytotoxicity might be associated with immune protection against *S. japonicum* infection. Induced and activated CTL to parasitic antigens during schistosomiasis have been previously investigated. Injection of DCs pulsed with OVApep in combination with SEA induced the priming of an OVA-specific CD8^+^ T-cell response, and Th1 and Th2 cells helped primary CD8^+^ T-cell responses and the establishment of long-lived CD8 memory [45]. Immunization with a single dose of rSm28GST was able to induce a reduction in worm burden, and resulted in the *in vivo* induction of a significant CTL activity in the spleen. Adoptive transfers of Sm28GST-specific CD8^+^ T cells reproduced the protective effect obtained with the recombinant molecule [46]. These studies demonstrated that the genes activation related to cytotoxicity and CD4^+^ T cells was associated with the protective immunization against schistosomes. Furthermore, NK cells and CTL also produce IFN-γ in pigs, which may promote the killing of the schistosomule indirectly.

In conclusion, the UV-attenuated cercariae vaccine generated significant protection in pigs against *S. japonicum*, and the high level protection were correlated with a strong IFN-γ, IgG2 antibody response and upregulated expression of some cytotoxic genes. Although the pig model could not completely reflect the host/parasite relation in human, the data gave us confidence to continue the task of converting it to recombinant antigens vaccine formulation. Findings from this study implied the molecular mechanisms underlying high level protection induced by UV-attenuated cercariae vaccination and would lead us to a rationale for the search of antigenic epitopes for the stimulation of specific T cells, and significant antibody isotype as well as cytotoxicity. The remained challenge is the identification of key molecules to mimic and accelerate the process that leads to resistance.
